# Stevens–Johnson syndrome/toxic epidermal necrolysis associated with zonisamide

**DOI:** 10.1002/ccr3.1288

**Published:** 2017-12-20

**Authors:** Karina L. Vivar, Kimberly Mancl, Lucia Seminario‐Vidal

**Affiliations:** ^1^ Department of Dermatology and Cutaneous Surgery University of South Florida Tampa Florida

**Keywords:** Drug rash, pediatric, Stevens–Johnson syndrome, toxic epidermal necrolysis, zonisamide

## Abstract

This report highlights zonisamide as a potential cause of serious cutaneous reactions as well as its cross‐reactivity with other sulfonamides. Here, we present a case of SJS‐TEN due to zonisamide, which was effectively treated with IVIg. Subsequently, the patient was transitioned to levetiracetam for seizure control.

## Introduction

Zonisamide is a second‐generation antiepileptic drug (AED) that was approved by the U.S. Food and Drug Administration in 2000 as an adjunctive therapy for partial seizures in adults with epilepsy 1. It was first used in Japan starting in the 1970s as a psychiatric medication and later in Japan and Korea in the 1990s for treatment of seizures. As a synthetic 1,2‐benzisoxazole‐3‐methanesulfonamide, zonisamide is chemically related to atypical, second‐generation antipsychotic agents like risperidone as opposed to the first‐generation AEDs such as carbamazepine 2.

The use of zonisamide as an antiepileptic agent in both children and adults has been increasing after it was demonstrated to be as effective as first‐generation AEDs for the treatment of partial epilepsy 3. Zonisamide is also being used as off‐label monotherapy and adjunctive therapy for psychiatric disorders such as alcohol abuse, bipolar disorder, and binge‐eating disorder 4. Advantages of zonisamide, particularly in the pediatric population, include its once‐daily dosing (divided dosing can be used if needed), being relatively well tolerated, being unlikely to react with other medications, and not requiring serum level measurements 3, 5.

Here, we present the youngest patient with SJS‐TEN overlap syndrome secondary to zonisamide.

## Case Report

A 2‐year‐old African American girl with a past medical history of extreme prematurity born at 25 weeks of gestational age and seizure disorder was transferred to our hospital with a rash for 1 day, with associated conjunctival injection and crusting around the eye. The mother reported that the girl did not want her skin to be touched, possibly secondary to pain. The mother also reported fever, decreased appetite, and decreased energy. There was no prior history of a similar rash. The patient had no rhinorrhea, cough, or congestion. Her medication history was significant for a change from levetiracetam to zonisamide for seizure control about two and a half weeks prior to presentation. Neurology had recommended the change for improved seizure control and for improved adherence since zonisamide was once‐daily dosing. The patient did not have any other new medications including antibiotics within the past month prior to presentation.

**Figure 1:**
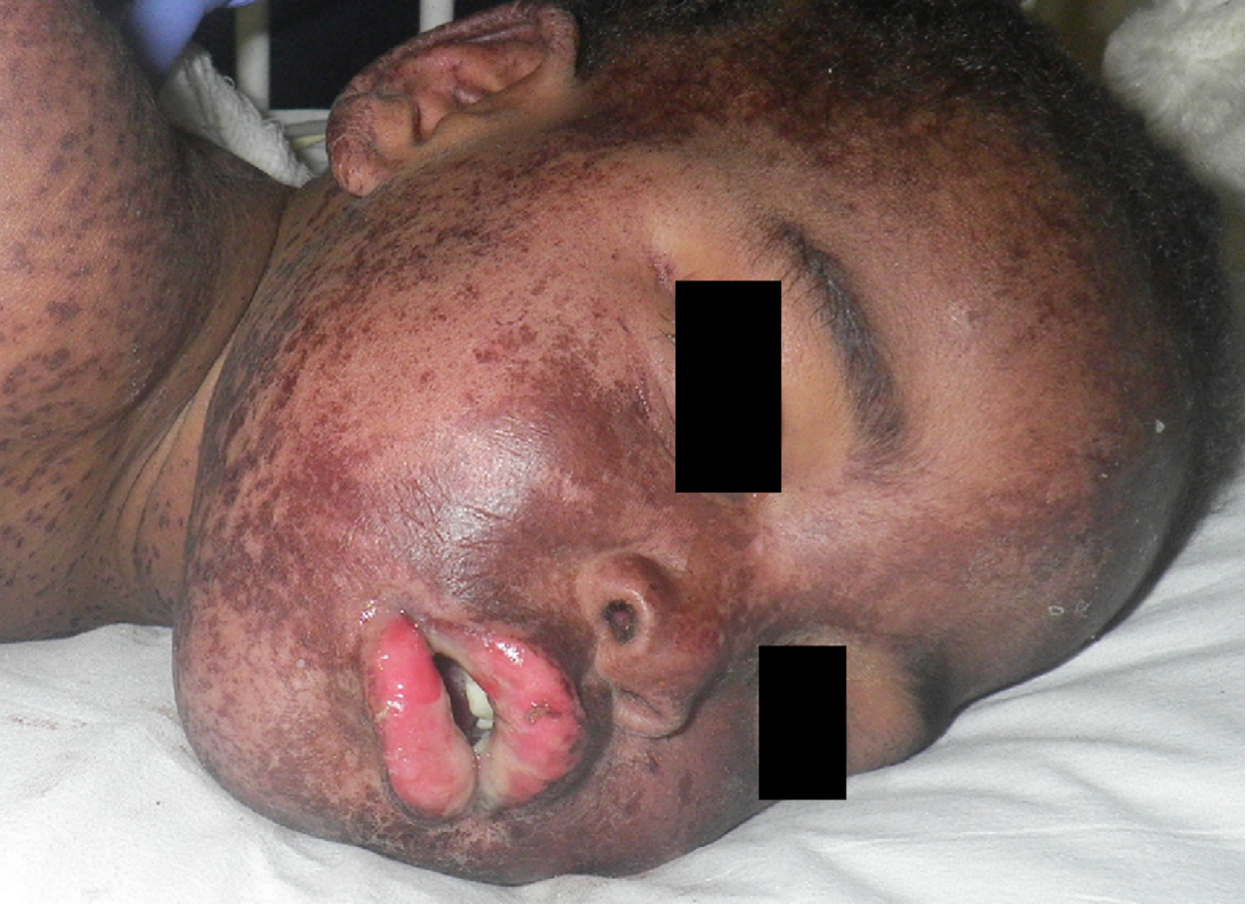
Clinical photograph of patient demonstrating dusky, erythematous macules, and papules as well as crusting and erosions of the lips.

**Figure 2:**
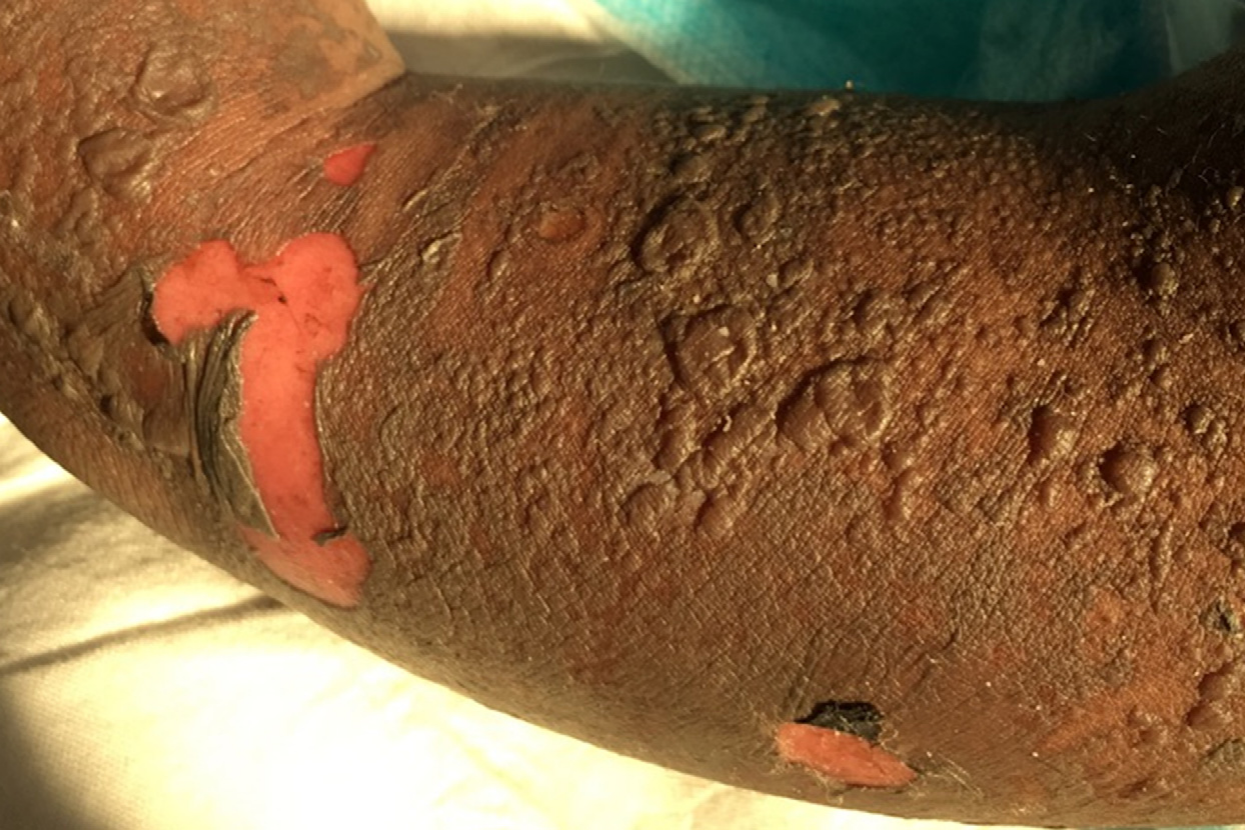
Clinical photograph of patient's bullae and full‐thickness epidermal desquamation.

On physical examination, the patient was afebrile and difficult to console. She had erythematous targetoid macules, papules, and plaques with dusky centers on her face, neck, chest, abdomen, back, bilateral upper extremities, and bilateral lower extremities involving 75% body surface area (BSA). Nikolsky sign was positive in 9% BSA upon presentation, and the rash progressed within 24 h to approximately 20% BSA Nikolsky positive. Tense bullae were noted on her face, arms, and legs with sloughing on the left upper arm at site of prior bandage. Mucosal lesions included erosions and crusting of her lips, conjunctival injection, and erythema and erosions of her labia minora (Figs 1 and 2).

**Figure 3:**
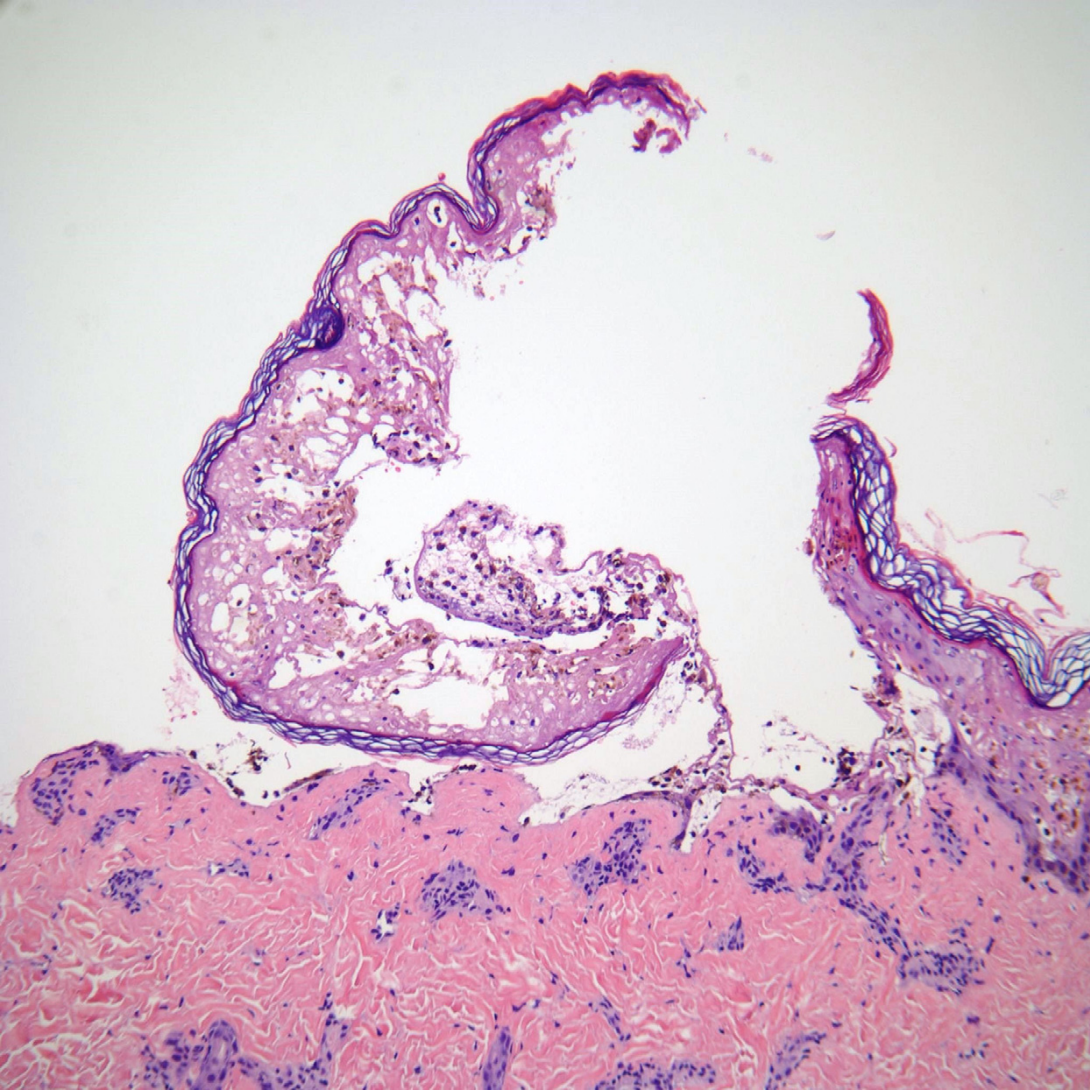
Biopsy specimen demonstrating full‐thickness epidermal necrosis, 100X.

Biopsies for frozen section and permanent pathology revealed full‐thickness epidermal necrosis with a pauci‐inflammatory infiltrate (Fig. 3). Herpes simplex virus and varicella zoster virus polymerase chain reaction tests from a bulla were negative. Laboratory studies revealed significant transaminitis with alanine aminotransferase (ALT) of 891 U/L and aspartate aminotransferase (AST) of 595 U/L. Alkaline phosphatase was also elevated at 304 IU/L. Complete blood count, electrolytes, and kidney function were within normal limits. Respiratory viral panel, hepatitis A immunoglobulin M (IgM) antibody, hepatitis B core IgM antibody, hepatitis B surface antigen, and hepatitis C antibody were negative. Serum urea, bicarbonate, and glucose levels were within normal limits. The patient was admitted to the pediatric intensive care unit with the diagnosis of Stevens–Johnson syndrome/toxic epidermal necrolysis overlap. Her severity of illness scale for toxic epidermal necrolysis (SCORTEN) was one 6.

Zonisamide was discontinued immediately, and intravenous immunoglobulin (IVIg, 1 g/kg/day) for 3 days was administered without complications. Neurology restarted the patient on levetiracetam for her seizure disorder. Her skin improved significantly over the next 2 weeks, and at the time of discharge, only mild crusting of her lips remained with all other cutaneous lesions healed. Her transaminitis resolved during admission as well.

## Discussion

To our knowledge, this is the second case of SJS‐TEN overlap syndrome associated with zonisamide reported in the United States 7.

Though usually well tolerated, common side effects of zonisamide include dizziness, sedation, headache, weight loss, decreased appetite, and decreased bicarbonate levels 3, 8. Rashes occur in up to 2% of patients treated with zonisamide and may be more common in patients receiving zonisamide in combination with first‐generation AEDs 9, 10. Serious cutaneous reactions include drug‐induced hypersensitivity syndrome, SJS, and TEN 9.

The incidence of SJS/TEN in the setting of zonisamide is not known. In the United States, the only reported case was a 16‐year‐old female adolescent with bipolar disorder who was switched from valproic acid to zonisamide secondary to weight gain. After 2 weeks of zonisamide, she developed a painful, bullous eruption and was diagnosed with TEN. This patient's mother reported being allergic to sulfa medications 7. In addition, one case of SJS was reported in a postmarketing surveillance study of 1938 patients treated with zonisamide who were followed for at least twelve weeks 8. Other details of the case were not published. Teraki et al. reported a 71‐year‐old man who was initially on valproate sodium for epilepsy and then started on zonisamide for seizure recurrence. Twenty‐three days after initiation of the zonisamide, the patient developed a targetoid, blistering eruption consistent with TEN. Interestingly, IgG titers and human herpesvirus 6 (HHV6) DNA levels in the patient's blood increased over the progression of his eruption, suggesting HHV6 reactivation 11. The other reported cases come from case series in Japan, where 13 patients with epilepsy were taking zonisamide and developed SJS/TEN. Three of the patients were taking zonisamide in combination with one aromatic antiepileptic such as phenytoin or carbamazepine. There were five females and eight males in this group. The median age of these patients was 52 years old, ranging from 6 to 71 years old. The average time to development of SJS/TEN was about 23 days 12.

In the two cases reported in detail in the literature and our case reported here, all three patients developed TEN within 2–4 weeks of starting zonisamide. Additionally, all cases were on zonisamide as monotherapy after prior exposure to another agent, either valproic acid or levetiracetam. Notably, our patient was the youngest to develop SJS/TEN in the setting of zonisamide. IVIg was used effectively in the case reported by Teraki et al. as well as in the present case. The third case by Majeres and Suppes did not report management.

Given the increasing use of zonisamide for neurological and psychiatric disorders, it has become important to determine the risk of potentially fatal cutaneous reactions such as SJS/TEN. Prior use of levetiracetam or valproic acid may play a role in predisposing to TEN. Family history of sulfa allergy may also be clinically significant 7. In Japan, where zonisamide has been prescribed for a longer period than in the United States, studies have shown that HLA‐A*02:07 may be a biomarker for susceptibility to zonisamide‐induced SJS/TEN 12.

Additionally, a study of in vitro cross‐reactivity between zonisamide and either sulfonamides or first‐generation AEDs underscores the chemical relatedness of zonisamide to sulfa medications. Lymphocyte toxicity assays (LTAs) from 20 patients with known DIHS to sulfamethoxazole demonstrated cross‐reactivity between sulfonamides and zonisamide. In contrast, LTAs from 20 patients with known DIHS to first‐generation AEDs did not cross‐react with zonisamide 9. Especially in light of recent case reports of HHV6 reactivation in SJS/TEN patients, classically thought of in DIHS, future studies may evaluate whether LTAs can also be used to predict SJS/TEN secondary to zonisamide 11, 13.

In sum, the risk of SJS/TEN associated with zonisamide appears to be low but real. The incidence of these serious and life‐threatening cutaneous eruptions due to zonisamide needs to be further investigated. The development of screening tests such as HLA typing and LTAs may also identify at‐risk patients. Clinicians should maintain high clinical suspicion in order to promptly discontinue the culprit drug and initiate appropriate intensive inpatient SJS/TEN management including diligent wound care, fluid and electrolyte management, and systemic therapies like steroids, intravenous immunoglobulin, or other immunomodulators. Importantly, clinicians should also be aware of the cross‐reactivity of zonisamide with other sulfa medications in order to counsel patients to avoid all sulfa medications in the future.

## Conflict of Interest

The authors have no financial or other conflict of interests to disclose.

## Authorship

KLV, KAM, and LS‐V: had full access to all of the data in the study, take responsibility for the integrity of the data and the accuracy of the data analysis, and involved in acquisition, analysis, and interpretation of data. KLV and LS‐V: involved in study concept and design. KLV and KAM: drafted the manuscript and provided administrative, technical, or material support. LS‐V: critically revised the manuscript for important intellectual content and supervised the study. None: performed statistical analysis and obtained funding.
